# Supplementary material for: Are carbon emissions trading and green financial instruments synergistic? -Comprehensive quantitative research based on content analysis
Source: PLoS One. 2024 Mar 7;19(3):e0298601. doi: 10.1371/journal.pone.0298601 (PMC10919601; doi:10.1371/journal.pone.0298601)
Supplement: S1 Data — (ZIP) [file pone.0298601.s001.zip › Data/地级市Z_F工作报告.zip!/╣π╢1⁄2/├╖╓▌╩╨/└┤╫╘╣1⁄2╓┌║┼í╛┬φ┐╦╩2╛▌═°í┐_2014.docx]

各位代表：

我代表梅州市人民政府向大会作报告，请予审议。请政协委员参议，请其他列席人员提出意见。

2013年工作回顾

2013年，在省委、省政府和市委的正确领导下，在市人大及其常委会和市政协的监督支持下，市政府坚持以邓小平理论、“三个代表”重要思想和科学发展观为指导，围绕“全力加快绿色经济崛起，建设富庶美丽幸福梅州”的核心任务，大力实施“一园两特带动一精”发展战略，全市经济社会发展呈现“稳中趋好”的态势，各项工作取得可喜成绩。

一、经济发展稳中向好

经济运行总体平稳。全市生产总值、地方公共财政预算收入、固定资产投资分别达800.01亿元、69.35亿元和280.50亿元，比上年分别增长11.1%、23.2%和27.5%，增速均超过全省平均水平。县域生产总值实现480.65亿元，增长12.2%；全市工业增加值241.24亿元，规模以上工业增加值172.62亿元，均增长14%。社会消费品零售总额450.18亿元、增长11.7%；外贸进出口总额17.63亿美元，增长17.3%。

实体经济发展步伐加快。制订实施产业振兴三年行动计划，出台系列扶持企业、产业发展壮大的政策措施，深入开展“暖企”行动，改造提升传统产业，加快培育新兴产业。全市骨干企业完成工业产值288.74亿元，增长12.4%，占全市规模以上工业总产值的51.5%。全市民营经济实现增加值474.55亿元，增长10.5%，占全市生产总值的59.3%。金融对实体经济的支持力度加大，全市本外币贷款余额547.53亿元，比年初增长20.6%。33家企业在广州或前海股权交易中心挂牌。国家水泥及制品质量监督检验中心、中国南方稀土产品交易中心落户我市。新增省工业类名牌产品11个。全市专利授权量1257件，增长38.3%。23家电商企业入驻梅州家乐电子商务产业园区。产业集聚发展平台加快建设，广州（梅州）产业转移工业园完成总体规划修编，园区建设面积扩展到36平方公里。各县（市、区）工业园区规划建设扎实推进。成功举办2013’世界客商大会。乡贤回乡投资兴业工程有效推进，全市引进合同项目268个，实际投资总额215.23亿元。

文化旅游实现新突破。成功竞得省山区（生态）旅游产业园区竞争性扶持资金。广东文化旅游特色区雁洋核心区建成一批新的景点和配套设施。“十村联动”美丽乡村加快建设。梅县区成为全国客家文化生态旅游知名品牌创建示范区。支持平远创建生态文明示范区和大埔建设客家文化生态保护区。开展 “我带大家游梅州”活动，“休闲到梅州，享受慢生活”品牌更加响亮。成功承办央视中秋晚会，优秀传统客家文化得到弘扬，梅州知名度和影响力进一步提升。雁洋申报国际慢城工作顺利推进。新增国家3A级旅游景区5家。全市旅游接待人数、旅游总收入分别增长39.7%和33.5%。

农业经济稳步发展。农业总产值、农业增加值分别达275.09亿元和164.71亿元，增长5.4%和5.6%。实施31个高标准基本农田建设项目，新增特色农业产业基地12.4万亩，建成国家、省农业标准化示范区26个。新增省级农业龙头企业17家、林业龙头企业8家，新增农民专业合作社1110个、家庭农场96家、省级名牌产品9个。创建了 “社区直供”新模式，梅州绿色健康农产品以专柜形式直供珠三角地区。“客都汇”农特产品电商信息网络平台正式上线。兴宁熙和湾、丰顺马山、大埔瑞山等20家休闲观光农业基地建设扎实推进。西岩乌龙茶获批国家地理标志产品原产地认证。市农产品质量监督检验测试中心通过省级计量认证。新一轮扶贫开发“双到”工作进展顺利，实施帮扶项目4161个，贫困户人均纯收入达4250元。蕉岭县农村综合改革试点工作有效推进。

二、城乡面貌变化明显

交通、水利等重点项目进展顺利。完成交通基础设施建设投资61.2亿元，增长48%。梅大高速三角至大麻段建成通车，兴华高速（含畲华支线）动工建设，梅大高速东延线、汕湛高速五华段、济广高速平兴段加快推进。梅汕高铁和鹰梅、浦梅铁路前期工作扎实推进。升级改造国道、省道134公里。开通梅县-深圳、梅县-台中航线，启动梅县机场迁建选址工作。总投资23.28亿元的268宗重点水利项目顺利推进。韩江（高陂）水利枢纽工程、粤电大埔电厂获国家核准建设。梅南水利枢纽工程、兴宁罗浮河引水工程等重点水利项目前期工作稳步推进。兴宁、五华、梅县等省级水利示范县建设进展顺利。实施江北老城区自来水管网改造。市清源水质净化中心二期工程通水投入运营。积极应对三次大洪灾，救灾复产、重建家园任务基本完成。投资10.85亿元的电网项目顺利实施。梅州（五华）抽水蓄能电站前期工作加快推进。

中心城区扩容提质和美丽城乡建设深入推进。国务院批准梅县撤县设区。嘉应新区发展总体规划获省政府批准实施。扎实推进江南新城、梅县新城规划建设，江南新城重点区域地段和芹洋半岛征地拆迁任务基本完成，安置区建设正有序推进，客商会展中心、梅州奥园、富港东汇城等一批城建项目动工建设。节约集约用地水平进一步提高，有效保障了项目用地需求。超额完成保障性住房建设任务。推进数字化城管建设。启动国家环境保护模范城市创建工作。饮用水源水质达标率达100%，城市空气质量优良率处于全省地级市前列。我市获“中国最具幸福感城市”和“中国十佳优质生活城市”称号。落实规范农村建房等政策措施，创建24个名镇名村、4个美丽乡村示范点。深入开展“绿满梅州”大行动，大力推进“一消灭三改造”工程，完成年度造林任务，建成生态景观林带310公里，森林覆盖率达73.55%。积极开展“清洁家园”创建活动，建设“万村绿”市级示范点24个。基本实现城乡生活垃圾处理全覆盖。环城高速公路亮化美化工程全面完成。淘汰落后产能693万吨，预计单位生产总值能耗下降3.91%。

三、民生和社会事业有新进步

人民生活持续改善。民生领域财政支出增长16.5%，占公共财政预算支出的75.7%。预计城镇居民人均可支配收入达20737元，增长10.9%；农民人均纯收入10148元，增长12.3%。全力办好十件32项惠民实事：全年培训劳动力13.8万人次，转移就业15.9万人次，新增城镇就业3.24万人，城镇登记失业率2.35%，有培训和转移就业意愿的新成长劳动力100%参加培训和转移就业；成功创建粤东西北地区首个教育强市，实施嘉应名师培养工程，一批知名专家学者和优秀博士、硕士研究生受聘到我市任教，学生饮用奶推广计划顺利实施，7000多名特殊困难学生免费喝上学生奶；嘉应歌剧院工程顺利推进，建成一批县级图书馆、博物馆、文化馆和镇村文化站（室）、电子阅览室，全面完成未通有线网络的农村地区广播电视“户户通”工程；城乡居民大病保险制度全面实施，建立3家平价医院，挂牌25家平价药店，对国家和省目标人群全面实施免费孕前优生健康检查；建成1003家食品安全流动示范点，市食品药品检验检测中心挂牌成立，建成1家“三星级”肉联厂，新型现代化屠宰场建设正在顺利推进；市中心枢纽汽车站动工建设，梅州城区初中生免费乘坐梅城公交车，完成新农村公路建设559.4公里；完成碳汇工程造林面积70.87万亩，环保能源发电项目有序推进，兴宁、平远、蕉岭、大埔、丰顺、五华生活垃圾无害化处理场完成年度建设计划任务，城乡环境进一步优化；完成66宗山区小型灌区改造工程、8宗中央和省财政小型农田水利重点县建设项目、82宗农村人口饮水安全工程；全市月人均低保补差水平、农村五保供养达到省定标准，免费为430户贫困家庭残疾人实施居家无障碍环境改造、330户贫困家庭残疾人提供居家康复服务，3家城市居家养老服务示范中心投入运营，改造一批乡镇敬老院，新增养老床位2945张；基本完成县级行政服务中心和市县网上办事大厅建设，网上申办事项占总事项的80.02%。

社会各项事业全面进步。扎实推进城乡教育均衡发展，学前教育、义务教育、高中教育、职业教育、特殊教育办学质量全面提高。 全市高中阶段毛 入学率93.63%。高考取得较好成绩。市120公共卫生应急指挥中心正式运行。稳定低生育水平，促进人口均衡发展。新增3处国家重点文物保护单位、50处市级文物保护单位和4个省级非遗项目。松口古街区被评为第五届 “中国历史文化名街”。我市被评为中国体育（足球）非物质文化遗产保护和推广城市。承办国际铁人三项、中国全地形车等大型赛事。国防教育、人民防空和民兵预备役建设扎实推进。“平安梅州”创建工作考评总分居全省第一。不断完善安全生产工作机制。我市成为广东唯一全域被确认为原中央苏区范围的地级市。发挥侨乡和海峡两岸交流基地优势，外事侨务、港澳和对台工作取得新进步。监察、审计、法制工作有新成效，民族宗教、人事编制、妇女儿童、统计、物价、供销、气象、残联、打私、档案、方志等工作取得新成绩。大力扶持发展社会组织，积极稳妥向社会转移职能。全面推进民情志愿服务工作，全市注册志愿者12.5万人。社会自律体系建设试点工作考核全省第二。依法成立广东雁洋公益基金会，为公益慈善事业发展注入新动力。支持社会组织举办首届客家文博会和客家文化创意产品设计大赛。

过去的一年，我们坚持推进法治政府、廉洁政府、创新政府和效能政府建设，健全重大行政决策机制，完善政府工作规则，强化重点领域、重点环节的权力运行监督，提高依法行政水平。市政府自觉接受市人大常委会依法监督和市政协的民主监督，共办理2件省人大建议、5件省政协提案和43件市人大建议、196件市政协提案，办复率100%。认真听取各民主党派、工商联、无党派人士及工青妇等人民团体意见。深入推进“六五”普法工作。加快政府职能转变，推进行政审批制度、企业登记审批制度、政府行政复议委员会试点等改革。清理压减市级行政审批事项185项。放宽市场主体准入门槛，全市新设立公司2720个，增长31.4%，各类市场主体总量达15万户。创新政务公开方式，依托人民网建立“梅州发布”制度，开展“微访谈”，6名市政府领导和8名市政府组成部门主要领导上线与网民沟通，共有1394.2万人次上网浏览、互动，推动一批民生热点问题的解决。“梅州发布”获评人民网2013年度“十大最具影响力政务微博”。建成“12345”政府服务热线平台，市政府网站在中国地市级政府网站互联网影响力综合排名第五。扎实开展党的群众路线教育实践活动前期工作，严格落实中央 “八项规定”，着力纠正“四风”，坚持立行立改，市政府会议文件简报均大幅减少，取消市级评比达标表彰活动，考核检查活动精简90%，政务接待进一步简化和规范。

各位代表！上述成绩的取得，离不开省委、省政府的关怀重视和市委的领导，离不开市人大和市政协的监督、支持，归功于全市人民同心同德、群策群力。在此，我谨代表市政府，向全市人民、中央和省属驻梅单位、驻梅部队，向所有关心支持梅州发展的海外侨胞、港澳台同胞和其他朋友们，表示最衷心的感谢并致以崇高的敬意！

在看到成绩的同时，我们也清醒地认识到，我市经济社会发展还存在不少困难和问题：一是经济总量小，人均生产总值水平低，要实现省提出到2020年达到全国同期平均水平压力很大；二是加快发展的交通“瓶颈”尚未突破，潜在的区位优势尚未突显；三是发展平台集聚能力弱，缺乏大项目支撑，优势产业未形成，经济发展的动力不足；四是中心城区辐射带动经济发展能力不强，县城和中心城镇承载产业和集聚人口的能力较弱；五是公务员队伍开拓创新、改革进取的锐气不足，等等。我们将正视这些困难和问题，切实采取措施加以解决。

2014年工作安排

各位代表！2014年，是全面贯彻落实党的十八届三中全会精神、全面深化改革的开局之年。做好今年的工作，必须重视跟踪分析和把握国内外形势的新变化，既要增强忧患意识，充分估计可能出现的困难和问题，更要看到发展机遇和有利条件，坚定加快发展的信心。党的十八届三中全会绘制了政治、经济、文化、社会和生态“五位一体”的改革总路线图，为全国各地经济社会转型升级注入了新的动力；国家正编制实施赣闽粤原中央苏区振兴发展规划，将有力推动包括梅州在内的原中央苏区加快发展；省委、省政府加快粤东西北地区振兴发展决定的深入实施，广州市全方位的对口帮扶，将有力推动梅州迎头赶上、振兴发展。我们须牢牢把握住这些重要的机遇和有利条件，切实增强加快发展的责任感和紧迫感，进一步解放思想，深化改革开放，把握任务重点，善于借势借力，努力走出一条加快发展、跨越发展的好路子。

今年政府工作的总体要求：深入贯彻落实党的十八届三中全会、中央经济工作会议、习近平总书记系列重要讲话和省委、市委全会精神，以胡春华书记、朱小丹省长来梅调研讲话精神为指导，以改革统揽全局，围绕“一个目标、三大抓手、两条底线”要求，深入实施“一园两特带动一精”发展战略，建设“三区三城”重点发展平台，扎实推进交通等基础设施建设，发展生态适宜型产业，做大实体经济，做强园区经济，推动中心城区扩容提质，统筹城乡建设，增创发展新优势，加快梅州振兴发展。

今年全市经济社会发展的主要预期目标：生产总值增长12%以上；地方公共财政预算收入增长15%以上；固定资产投资增长30%以上；单位生产总值能耗下降3.66%；人口自然增长率控制在9.3‰以内；城乡居民人均收入实际增长与经济增长基本同步。

重点是抓好七方面工作：

一、推动经济又好又快增长

我市经济发展水平离全国、全省平均水平还有很大差距，做大经济总量，夯实赶超全国平均水平的发展基础，是当前和今后一个时期的首要任务。

努力做大做强工业。全力配合广州市主导共建广州（梅州）产业转移园，成立产业投资基金，重点培育扶持成长性强、前景广阔的主导产业，推动园区扩能增效。结合嘉应新区建设，优化东升生态工业园规划，推进园区二期开发，推动电子信息、电子商务、现代物流及生物医药等产业向园区集聚。把握产业发展规律，深入实施产业振兴三年行动计划，建立领导干部挂钩支持重点产业发展制度，借助广州雄厚的产业基础和成熟的发展模式，大力推动传统优势产业的资源整合、增资扩产、技术改造。支持骨干行业、重点企业技改升级、扩能增效。加快培育电子电声、新型建材、生物医药、食品加工、特色陶瓷、新型环保等潜力产业，努力把电子信息、烟草、建材、电力、机电、矿产资源深加工等打造成产值超百亿元的支柱产业。开展质量强市，推进检验检测公共技术服务平台建设。

抓好文化旅游、健康养生产业发展。放大省扶持生态旅游资金效应，撬动民间投资，加快推进广东文化旅游特色区建设。重点抓好雁洋生态旅游产业核心区、平远生态旅游产业园、大埔客家文化生态保护区、丰顺温泉城等项目。充分挖掘古城镇、古村落、古民居蕴含的客家历史、人文内涵，加强保护和开发，策划包装一批客家“名人名居”文化景区。制定实施健康产业发展规划，引进有实力的企业进行整体开发，加快发展休闲度假、保健疗养、健康管理、运动休闲等产业，开发推广养生、长寿、体验等高端休闲旅游产品。推进旅游综合设施建设，抓紧完善景点景区的旅游服务功能配套。加快智慧旅游试点城市建设。继续开展 “我带大家游梅州”活动。加强旅游产业的整体策划营销，增强市场吸引力和竞争力。大力发展文化创意产业，通过市场化运作扩大客家文博会影响，使之成为做大客家文化创意产业的重要平台。

努力扩大消费需求。坚持扩大内需稳定外需，大力发展电子商务等现代服务业。培育发展电商龙头企业，建设电子商务产业园，推动电商集聚发展。加强对互联网新技术的应用，建设专业镇电子商务平台，积极组织企业“上网触电”。构建梅州电子商务产业体系，大力推动电子商务与特色农产品、客家文化、生态旅游等特色产业融合发展。推动电商校企合作，培养引进电商人才。规划建设一批大型专业市场、商业街区和物流园区。建立公共物流信息平台，建设第三方物流基地和物流仓储中心。鼓励有条件的大中型企业从企业物流发展成为专业物流企业，实现优势产业与物流业的联动发展。积极拓展住房、养老、医疗保健等服务领域，培育新兴消费热点。落实带薪休假制度，提升居民消费水平。创新信息服务产品和模式，支持4G网络建设和市场拓展，扩大信息消费。积极拓展国际市场，促进电子信息、陶瓷、电声、工艺等出口产品向高端延伸，逐步形成以技术、质量、品牌、市场和服务为核心竞争力的出口优势。

全力发展县域经济。依托各县县城，大力推进各县（市、区）生态工业园的开发建设。因地制宜，大力培育发展三个以上主导产业和一批具有较强带动能力的龙头骨干企业，推动形成产业集聚功能，增强县域经济支撑力。市、县两级政府要加大对专业镇的扶持力度，支持每个县（市、区）做强2-3个专业镇，进一步打响一批县域特色品牌。认真抓好原中央苏区县有关扶持政策的跟进落实，争取更多的项目和资金支持。

大力发展民营经济。全面落实中央和省委、省政府对民营经济的各项扶持政策，坚持机会平等、权利平等、规则平等。实行“负面清单管理”，清理和废除对非公有制经济各种形式的不合理规定，清除各种隐性壁垒，制定和落实非公有制企业进入特许经营领域的具体办法。保障民营企业的合法权益，真正落实对民营经济的“国民待遇”。鼓励民营企业围绕主导产业和产业链配套生产，打造珠三角先进制造业的配套基地。引导支持中小企业拓宽融资渠道，扶持一批民营企业进入区域股权交易中心融资发展，推动10-20家企业在“新三板”挂牌交易，培育1-2家企业上市。继续实施客商培训计划，提高民营企业家的素质和能力。

提高招商引资实效性。密切与广州的沟通联系，建立两市招商引资信息共享平台，积极承接珠三角等发达地区产业转移项目和特色资源项目，引进大集团、大财团和社会资本参与重点项目和工业园建设。充分发挥港澳台等海内外乡贤众多的优势和商会、行业协会的作用，注重产业链招商、专业招商，突出以商引商，力争引进一批潜力大、增长性强的企业群落。严守环保底线和效益底线，健全进园企业进入和退出机制，提高园区容积率、土地利用率和税收水平。用好低丘缓坡未利用地开发试点等政策，盘活土地资源，保障项目用地。

二、致力抓好交通等重要基础设施建设

加强交通等基础设施建设，构建发达的交通网络，是突显梅州区位优势、吸引投资和集聚产业、加快发展的关键。

突破一批交通重点项目建设。完成梅大高速（大麻至三河段）及其东延线建设。全力推进汕湛高速五华段、济广高速平兴段、兴华高速（含畲华支线）建设。力争动工建设梅汕高铁和东环高速公路。扎实做好鹰梅、浦梅铁路、粤东城际轻轨丰顺延长线和大潮（含梅漳支线）、梅平、大丰华高速公路前期工作。完成205国道梅州段改造提升工程。争取动工建设9个国省道改造项目。改造提升县乡公路和重点景区旅游公路路网。加快推进梅州市中心枢纽汽车站建设。完成梅县机场航站楼改造，增开航线加密航班，大力推进梅县机场迁建前期工作。完成韩江航道整治工程。

着力抓好水利能源等重大项目建设。全面推进278宗重点民生水利工程建设，动工建设韩江（高陂）水利枢纽工程辅助配套项目，做好梅南水利枢纽工程前期工作。完成兴宁、五华、梅县等省级水利示范县年度建设任务，大力推进中央和省财政小型农田水利重点县建设。积极实施32宗小流域综合治理、中小河流治理和水库除险加固工程。完成梅州城区供排水体制改革，稳步实施城区水务合作项目。加大力度推进粤电大埔电厂、梅县清洁能源发电项目和梅州 （五华）抽水蓄能电站建设。完成农村电网改造升级工程。

加强信息基础设施建设。深入实施信息产业行动计划，加快推进广东电信梅州互联网数据中心 （IDC）、云计算电子政务数据(容灾备份)平台等基础设施建设，实施宽带普及提速工程，促进“三网融合”。运用信息化提升工业化，推进农业产业化和新型城镇化建设。大力发展信息公共服务平台，积极发展信息服务业，推进物联网、云计算示范应用，建设智慧城市，通过信息技术提升交通、医疗、教育和城市管理等领域的管理水平。延伸电子政务网络到城市社区和农村。

三、积极稳妥推进新型城镇化

梅州城镇化水平目前离全国、全省平均水平还有较大差距，提升城镇化还有很大空间。同时，我市经济欠发达，县城和中心镇集聚带动能力弱，创业、就业机会还偏少。推进城镇化必须遵循规律、因势利导，建立人流、物流、资本流和信息流等要素集聚机制，把城镇化建立在坚实的产业基础之上，防止摊大饼式扩张，避免“有城市无产业”、“有速度无质量”、“半城镇化”和“房地产化”等系列问题。

推动中心城区扩容提质。根据梅县撤县设区的新变化，抓紧编制梅州市中心城区总体规划，实现梅县区城市规划与原梅州城区的融合。以江南新城和梅县新城建设为重点，加快推进嘉应新区建设。大力推进江南新城骨干道路、剑英湖和芹洋半岛品牌战略发展区建设，拉开城市新区道路架构。坚持产城联动，合理布局新区产业发展和城市功能配套。强化经营城市理念，逐步实施城市土地一级开发，募集城市建设资金。探索建立多元可持续的资金保障机制，争取发行市政债券，拓宽城市建设融资渠道。推进城中村改造，优化江北古城，努力改善江北人居环境。

加强县城和中心镇规划建设。完善市域城镇体系规划，优化城镇化布局和形态，积极推进各县城和各中心镇的规划建设。坚持集约节约用地，提高城镇建设用地利用效率，力促镇村规划覆盖率达85%，建设一批有历史记忆、地域特色、文化传承的美丽城镇。鼓励社会资本参与城市公用设施投资运营，提高城镇公共服务水平。积极探索按居住地登记户口的户籍管理制度，鼓励本地农业人口就近落户城镇。加强进城务工人员职业培训和保障随迁子女享有义务教育权利。

四、坚持不懈抓好三农工作

深入落实中央一号文件精神，进一步强化农业的基础地位，发展农业生产，改善农村条件，深化农村综合改革，释放农村活力，努力促进农业持续增效、农民持续增收和农村稳定和谐。

提升精致高效农业发展水平。全面落实强农惠农富农政策。大力推进高标准基本农田、现代农业示范区建设。开展粮食稳产增产行动，确保粮食安全。大力引进良种良法，完善农业技术推广网络，加大农业机械研发、示范、推广力度。注重农业标准化建设，抓好特色农产品的重点规划，策划经营“中国长寿之乡”品牌，提升梅州金柚、平远慈橙、嘉应茗茶等品牌价值，开发更多绿色健康无公害农产品。支持台湾农民创业园、市绿色生态水产创新示范园建设。发挥农业协会作用，提高农业社会化服务水平。新组建一批“农超对接”的农民专业合作社。完善农产品流通体系，搭建农产品物联网和电子商务平台，加快推进大型农产品物流园配送中心等建设。支持供销合作社加强农村现代流通网络和农产品批发市场建设。强化农产品质量和食品安全监管，建立完善的疫情预警预报机制和应急处置机制。推动中国林业产权交易所在梅州设立合作机构，促进林业产业发展和生态补偿制度建设。

推进美丽乡村建设。以点带面推进幸福村居创建和名镇名村示范村建设。以治理垃圾、污水为重点，开展村庄人居环境整治。以县（市、区）为主体，为农户提供舒适、实用、美观的户型设计服务，引领农民规范建房。大力发展农村公共事业，促进邮政、通信、客运、物流、物业管理等向农村延伸。动员社会力量参与美丽乡村建设。扎实做好新一轮扶贫开发“双到”工作，因地制宜发展特色经济项目，促进贫困村、贫困户稳定脱贫。

调动农民积极性。广辟农民增收渠道，切实保障农民合法权益。积极推进新型农业经营体系建设，鼓励农民参与发展专业庄园、家庭农场等新型农业经营主体。盘活农村土地资源、金融要素，推进农村土地确权，加快健全土地经营权流转市场，完善县镇村三级服务和管理网络，鼓励有条件的农户流转承包土地的经营权，允许承包土地的经营权向金融机构抵押融资，推动农村土地集约化、规模化、产业化经营。因地制宜、合理保障农民的住房、社保、就业培训，确保被征地农民长期受益。深入推进农村精神文明建设，倡导移风易俗，培养良好道德风尚，提高农民综合素质。加强对农村留守儿童、留守妇女、留守老人的关爱和服务，健全农村治安防控体系，发挥司法调解、人民调解的作用，维护农村社会和谐安定。

五、大力推进生态文明建设

梅州是生态发展区，优势在于有好的生态、潜力也在于好的生态。要树立“生态立市”的理念，动员全社会、全民参与营造良好生态，让爱护环境、呵护自然、享受生态成为风尚。

狠抓生态建设和环境保护。深入推进新一轮“绿满梅州”大行动，全面实施森林碳汇、生态景观林带、森林进城围城、乡村绿化美化四大工程，完成“五沿”范围内林业重点生态工程建设任务，加强森林资源管护，构建森林生态屏障体系。动员全市力量，用两年时间打好“一消灭三改造”歼灭战，今年全力消灭宜林荒山26万亩，改造疏残林、纯松林、低效速生林60万亩。完成省立绿道建设任务，促进绿道网延伸升级。扎实推进国家环保模范城市创建。大力开展“南粤水更清”行动，加强中型水库水质管理和控制，规划建设中心镇污水处理设施，开展沿河排污企业整治，保护水源水质。加强重金属污染防治，推进土壤污染治理。深入实施城乡环境综合整治工程，力争全市城镇生活垃圾无害化处理率达85%以上。积极做好国家卫生城市复检各项工作，推进省卫生镇村创建。争取成为国家生态文明先行示范区。

积极推动节能减排和发展循环经济。加强大气污染和噪声综合整治，严格管控工业企业废气、建筑扬尘、机动车尾气排放，治理公共场所经营性餐饮污染。完成淘汰落后水泥、冶炼产能年度目标任务。加大高效照明产品、新能源汽车等节能产品推广力度。发展绿色节能建筑，扎实推进国家可再生能源建筑应用示范市建设。引导、扶持企业按循环经济模式进行生产经营，加大清洁生产技术改造实施力度，培育一批清洁生产企业。积极推进工业园创建国家和省级循环经济清洁生产示范园区。开展节能减排全民行动，倡导绿色低碳生活方式。

加快生态文明制度建设。编制生态文明建设规划纲要，全面实施主体功能区规划，划定生态保护红线，落实用途管制。积极探索与生态文明建设相适应的体制机制建设，实行最严格的生态保护、责任追究制度，实施企事业单位污染物排放总量控制制度。严格执行环境影响评价制度，强化环评管理。推进公众广泛参与的环保制度建设，及时公布环境信息，加强社会监督。

六、切实抓好民生保障和社会公共治理

人民群众安居乐业、社会和谐有序是一个地区文明进步的综合体现。要坚持把改善民生、促进社会和谐作为提升政府履职能力和水平的重要抓手，让人民群众共享改革发展的成果。

增加城乡居民收入。坚持高质量促进劳动力转移就业。围绕市场取向，做优职业技术教育，加强新成长劳动力技能培训。逐步完善政府扶持创业的配套政策措施，鼓励和支持自主创业。多形式扩大就业，多渠道增加工资性收入。落实最低工资制度，保障劳动者合法权益。完成培训城乡劳动力8万人次，转移就业10万人次。新增城镇就业3万人，城镇失业人员再就业2.6万人，城镇登记失业率控制在3%以内，全年促进创业2500人。

完善社会保障体系。坚持社会保障与经济发展同步提升，继续加大扩面征缴力度，完善“五险”市级统筹机制。积极推进保险制度改革，构建社会保险与商业保险互相促进、覆盖城乡的社会保障体系。建立健全以医疗、养老、最低生活保障、灾民救助和“五保”供养为基础，以教育、住房、法律等专项救助为辅助，其他救助和社会帮扶为补充的社会救助体系。提高失独家庭补助标准。实施农村独生子女家庭政府津贴政策。

提升公共服务水平。巩固提升教育“创强”成果，力争实现教育强县全覆盖。基本普及学前教育，加强标准化学校建设，推进义务教育发展基本均衡县工作。提升普通高中教育水平，筹建一所高等职业技术学院。支持嘉应学院实施省“创新强校”工程和东校区建设，以及医学院临床医学本科专业开办工作。研究出台支持民办学前教育政策的具体实施细则。继续实施学生饮用奶计划。扶持基层、社区和农村公共文化发展，扎实推进广电民生工程建设，繁荣群众文化，鼓励精品创作。推动客家博物馆争创国家二级馆。动员和整合各方力量，建设一批小微型艺术馆、收藏馆，支持带动民间艺术和民间收藏活动。深化县级公立医院综合改革，鼓励合作办医、民营资本办医。启动“奶粉进药店”试点工作，用最严格的药品管理办法监管质量。推进镇、村两级便民服务中心（服务站）建设。增开、延长公交线路。积极适应计生政策调整，促进人口均衡发展。推进妇女事业全面进步和儿童健康成长。

创新社会治理。大力开展社会工作示范镇、示范社区、示范村创建活动。实施社会创新项目，建设“省社会创新实验基地”。加大力度培育发展行业协会、商会、公益慈善、城乡社区服务、志愿服务等社会组织，强化行业自律管理，让社会组织承担更多公共服务。扎实推进“平安梅州”建设。完善综治信访维稳平台、应急指挥平台建设。继续推进打假治劣工作。铁腕整治安全生产，遏制较大以上安全事故发生。加强互联网管理，善用各类媒体，营造和谐稳定的社会环境。完成第三次全国经济普查工作。统筹推进国防动员、人防、双拥、气象、防灾减灾、民族宗教和政府法制、人事编制以及统计、方志、档案等各项工作上新水平。

七、积极推进各项改革

今年是全面贯彻落实《中共中央关于全面深化改革若干重大问题的决定》的第一年。我们要按中央和省委的总体部署，紧密结合梅州实际，以改革增创梅州发展新优势。

凝聚改革共识。统一广大干部群众的思想和行动，寻求最大公约数，引领干部群众参与改革、支持改革。处理好政府与市场的关系，着力把市场化改革引向新的广度和深度，使市场在资源配置中起决定性作用。更好发挥政府作用，把握好深化改革和加快发展的关系，把改革的力度、发展的速度和社会可承受的程度统一起来。以自我革新的勇气和胸怀，全面深化各项利益改革，努力增创营商环境、创新驱动发展、开放型经济、产业竞争、文化和生态的新优势。

务实推进改革。坚持问题导向，敢于打破利益藩篱，下大力气解决群众最急、最需、最盼的问题，稳妥有序、协调推进改革。发挥经济体制改革的牵引作用，统筹推进经济、文化、社会领域和城乡统筹、生态文明体制等改革，促进公共资源均衡配置。加快政府职能转变，全面正确履行政府职能，加强市、县（市、区）政府公共服务、市场监管、社会管理、环境保护等职责，支持和促进镇政府和街道办事处强化服务群众的职能，提升服务群众的能力和水平。进一步深化行政审批制度改革，继续精简行政审批事项。对保留的审批项目，要规范和优化审批流程，推行实时在线监察，建立“告知承诺”工作机制。大力推进政府向社会组织购买公共服务，制定实施政府向社会转移职能事项目录，鼓励社会资本参与住房、教育和医疗等社会事业建设。全面开展商事登记制度改革，培育发展市场主体，激发市场活力。加快建设社会信用体系和市场监管体系，努力营造国际化、法治化营商环境。加大投融资体制改革，落实“非禁即入”投资政策，支持非公有制经济参与平等竞争，探索公共投资领域引入社会资本，在公共资源配置中进一步引入竞争机制；推进投资项目审批制度改革，推行并联审批；推动融资模式向企业主导、“借用还”一体化转型。组建市公共资源交易中心，建设集政府采购、工程建设、产权交易和土地出让于一体的公共资源交易平台。顺应市场化形势，以规范决策经营、资产保值增值、提高企业效率、增强企业活力为重点，务实推进国有企业改革。鼓励非公有制企业参与国企改革，鼓励发展非公有资本控股的混合所有制，鼓励有条件的私营企业建立现代企业制度。深化财税金融制度改革，改进预算管理制度，提高预算透明度。严格政府举债程序，建立债务风险防控和预警机制。加强地方金融生态环境建设，防范和化解金融风险。加快建设农村金融改革创新综合试验区，探索农村综合金融要素市场建设，支持成立民营和村镇银行。总结推广蕉岭农村综合改革经验，新增两个县开展农村土地承包经营权确权登记试点工作。

各位代表！加强自身建设，是政府正确履行职能和提升综合服务能力的重要保证。市政府将把加强自身建设，贯穿在依法履职的全过程，努力建设人民满意的政府。坚持依法行政。贯彻落实法治广东建设五年规划，加强法治政府建设，推进依法治市。建立政府采购法律服务制度。严格执行市人大及其常委会的决议、决定，办好人大议案、代表建议和政协提案，主动接受人大、政协监督和舆论监督。坚持求真务实。结合开展以“为民、务实、清廉”为主题的党的群众路线教育实践活动，着力解决群众普遍关注的热点难点问题。完善实体办事大厅、网上办事大厅和“12345”政府服务热线三大平台建设，完善提升“梅州发布”微访谈活动，拓宽市政府和社会的沟通渠道。加强公务员教育管理和能力提升，依法公开政务信息，提高决策透明度，以政务诚信促进社会诚信建设。坚持廉洁从政。坚决落实中央八项规定，切实提高政府运作效率和效益。严格执行党风廉政建设责任制，遵守廉洁从政有关规定。加强行政效能监察和审计监督，推进市电子纪检监察综合平台建设，强化财政资金使用、重大投资项目、工程招投标、政府采购等重点领域监管。深入推进廉洁城乡建设，营造风清气正的干事创业环境。

各位代表！提高基本公共服务均等化水平，让经济社会发展成果更广泛地惠及全市人民，是我们的不懈追求。今年，在继续办好去年实事的基础上，我们将全力办好以下十件民生实事。一是保障底线民生。各县（市、区）城镇低保月人均补差水平提高到不低于334元、农村不低于148元；农村五保年供养标准提高到不低于当地上年度农村居民人均纯收入的60%；低保户基本医疗保险政策范围内住院自负医疗费用的救助比例提高到70%以上；城乡居民养老保险基础养老金人均标准下半年由65元/月提高到80元/月；残疾人生活津贴标准由100元/年提高到600元/年，重残护理补贴标准由600元/年提高到1200元/年。二是提高社会保障水平。职工养老保险的企业退休养老金提高10%，城乡居民医保每人年补助标准由280元提高到320元，城镇职工医疗保险年最高支付限额由30万元提高到33万元，城乡居民医疗保险住院报销比率提高5%；职工医疗保险、城乡居民医疗保险与广州6家医院实现联网即时结算；对民办养老机构（含公办民营）入住老人每月每床给予100元运营补贴。三是促进教育均衡发展。动工建设8所实验学校 （幼儿园）；全市义务教育标准化学校覆盖率提高到80%；城乡免费义务教育生均公用经费补助标准小学由750元提高到950元、初中由1150元提高到1550元。四是提高基层医疗卫生服务水平。人均基本公共卫生服务经费标准由30元提高到35元以上；实施偏远乡镇卫生院在编人员岗位津贴补贴政策；建设各县（市）120公共卫生应急指挥中心；为每个乡镇卫生院配置“五个一”医疗设备；提高地中海贫血筛查率，重型地中海贫血发生数下降50%。五是提升人居环境。完成各县（市、区）污水处理厂二期工程建设；改造185台重过载配电变压器和766个低电压台区，改善6.18万户居民用电质量；完成梅州城区90公里6～8米道路（巷道）路灯照明建设；实现城市建成区数字化城市管理全覆盖；完成市级能源管理中心建设；开工建设保障性住房、棚户区改造住房共9068套（户），完成13227户农村低收入住房困难户住房改造。六是开展助困扶残。为全市城乡低保户和农村五保户家庭每户每月提供15度免费用电；为1000名视力、1000名听力、1000名肢体贫困残疾人免费提供康复服务；分两年为视障人员免费配发读屏软件。七是关爱救助精神疾病患者。规划迁建梅州市第三人民医院（精神病专科医院）；给1万名重性精神疾病患者免费发放药物，免费收治500名贫困重症精神疾病患者。八是实施交通便民工程。动工建设客都大桥；完成新农村公路路面硬化改造工程400公里；实施梅州城区“治堵工程”，改造五个交通环岛，修复渡江津桥，打通四条城区市政道路。梅州城区增加LNG新能源公交车50辆，梅县区新增出租车100辆。九是丰富群众文化体育生活。建设梅江群众文化长廊和一批公园绿地歌舞台；新建30个农村文化俱乐部试点；完成县级多厅数字影院全覆盖工程建设；建成乡镇（街道）公共电子阅览室12个，完善349个行政村（社区）文化室设备；全市建成10个以上社区体育小公园、10个以上乡镇农民健身工程、12个标准足球场和其它体育设施。十是实施绿满梅州工程。完成森林碳汇重点生态工程建设50.85万亩；全市规划新建21个城区周边森林公园和湿地公园；建成200公里生态景观林带；建设24个“乡村绿化美化工程”市级示范点。

各位代表！新的一年，我们的机遇和挑战并存，让我们在省委、省政府和市委的领导下，抢抓机遇，改革创新，凝聚力量，攻坚克难，为加快振兴发展，建设富庶美丽幸福梅州而努力奋斗！
